# Low-Level Vibrations Retain Bone Marrow's Osteogenic Potential and Augment Recovery of Trabecular Bone during Reambulation

**DOI:** 10.1371/journal.pone.0011178

**Published:** 2010-06-17

**Authors:** Engin Ozcivici, Yen K. Luu, Clinton T. Rubin, Stefan Judex

**Affiliations:** Department of Biomedical Engineering, Stony Brook University, Stony Brook, New York, United States of America; Ohio State University, United States of America

## Abstract

Mechanical disuse will bias bone marrow stromal cells towards adipogenesis, ultimately compromising the regenerative capacity of the stem cell pool and impeding the rapid and full recovery of bone morphology. Here, it was tested whether brief daily exposure to high-frequency, low-magnitude vibrations can preserve the marrow environment during disuse and enhance the initiation of tissue recovery upon reambulation. Male C57BL/6J mice were subjected to hindlimb unloading (HU, n = 24), HU interrupted by weight-bearing for 15 min/d (HU+SHAM, n = 24), HU interrupted by low-level whole body vibrations (0.2 g, 90 Hz) for 15 min/d (HU+VIB, n = 24), or served as age-matched controls (AC, n = 24). Following 3 w of disuse, half of the mice in each group were released for 3 w of reambulation (RA), while the others were sacrificed. RA+VIB mice continued to receive vibrations for 15 min/d while RA+SHAM continued to receive sham loading. After disuse, HU+VIB mice had a 30% greater osteogenic marrow stromal cell population, 30% smaller osteoclast surface, 76% greater osteoblast surface but similar trabecular bone volume fraction compared to HU. After 3 w of reambulation, trabecular bone of RA+VIB mice had a 30% greater bone volume fraction, 51% greater marrow osteoprogenitor population, 83% greater osteoblast surfaces, 59% greater bone formation rates, and a 235% greater ratio of bone lining osteoblasts to marrow adipocytes than RA mice. A subsequent experiment indicated that receiving the mechanical intervention only during disuse, rather than only during reambulation, was more effective in altering trabecular morphology. These data indicate that the osteogenic potential of bone marrow cells is retained by low-magnitude vibrations during disuse, an attribute which may have contributed to an enhanced recovery of bone morphology during reambulation.

## Introduction

The removal of weight-bearing from the skeleton as a consequence of spaceflight, bedrest, paraplegia, or aging adversely affects the mass and architecture of trabecular bone [1], [2]. Unfortunately, full recovery of skeletal tissues upon reambulation may not be possible [3], increasing the risk of traumatic and atraumatic fractures and, ultimately, compromising quality of life [4]. Failure of the bone structure to recover on reambulation may in part be caused by the collapse of the osteogenic potential of bone marrow cell populations during disuse. Without relevant mechanical signals, marrow stromal cells with the potential to become bone cells may instead die or commit to other cell lineages such as adipocytes [5]–[7]. As a consequence, a reduced or distracted niche of osteogenic cells may not be capable to effectively rebuild the intricate skeletal morphology upon the reintroduction of regulatory signals associated with load-bearing [8].

Consistent with the importance of mechanical signals to maintain the osteogenic potential of bone marrow cells, superposition of exogenous mechanical signals onto normal daily activities can enhance bone at both the cellular and tissue levels [9]–[11] with exercise promoting osteoblastogenesis and inhibiting adipogenesis [12]. Despite the various benefits that exercise provides, many exercise-based interventions have been ineffective in stemming tissue deterioration during disuse [1], [13], [14] or to fully recapture bone mass upon reambulation [1], [15].

Exercise typically imposes a limited number of loading cycles at relatively high magnitudes (>1200 microstrain) and low (<10 Hz) loading frequencies [16], [17]. Functional daily activities, however, subject the skeleton to a much greater spectrum of loading magnitudes, frequencies and cycles, including high-frequency signals induced by quasi-isometric muscle activity [18], [19]. As bone can sense and respond to high-frequency mechanical signals, even if applied at extremely low magnitudes [20], [21], it is conceivable that that these mechanical signal components are critical to the retention of cellular and tissue homeostasis. Consistent with this hypothesis, the decline in trabecular bone formation rates during disuse can be rescued by brief applications of low-magnitude whole body vibration, but not by similar periods of normal weight bearing [22]. These physical signals retain their osteogenic influence even when the mode of application virtually eliminates extracellular matrix deformations [23], [24]. It is therefore possible that high-frequency mechanical stimuli are sensed directly by cells within the bone marrow to initiate a cascade of events promoting the population of mesenchymal cells and biasing their differentiation towards osteoblastogenesis.

In the healthy, physically active skeleton, low-magnitude whole body vibrations can potentiate bone's anabolic responsiveness by biasing the differentiation and proliferation of mesenchymal stem cells in the marrow towards a musculoskeletal lineage [25]. The importance of these mechanical signals in preserving the viability of stromal cells in the bone marrow environment during disuse is unknown. Here, we tested whether specific bone marrow cell populations as well as trabecular bone morphology can benefit from the application of low-level whole body vibrations during disuse and reambulation. In the second phase of this study, it was investigated whether trabecular bone recovery during reambulation can be augmented more effectively by applying mechanical signals only during disuse or only during reambulation.

## Materials and Methods

### Experimental design

All procedures were reviewed and approved by the Institutional Animal Care and Use Committee (IACUC). Seven-week old male C57BL/6J (B6) mice were used for all phases of the study (n = 108 total). At this age, trabecular bone mass has peaked in this specific inbred mouse strain [26] even though the overall skeleton has not reached peak bone mass and still undergoes modeling [27]. Three groups of mice (n = 24 each) were subjected to hindlimb unloading *via* the Morey-Holton model [28] for 3 w, depriving the hindlimbs of gravitational loading. The first group (HU) was subjected to hindlimb unloading only. In the second group (HU+VIB), HU was interrupted for 15 minutes per day (min/d), 7 days per week (d/w) by the application of high-frequency (90 Hz), low-magnitude (0.2 g, where 1 g  =  earth's gravitational field  = 9.8 m/s^2^) whole body vibrations. During this 15 min period, mice were allowed to roam freely on the vertically oscillating plate. The third group (HU+SHAM) was treated identically to HU+VIB mice except that the vibrating plate on which they ambulated for 15 min/d was inactive. A group of age-matched control mice (AC, n = 24) were individually housed in normal cages for the duration of the study to provide a reference for age-related changes in the skeletal phenotype.

At the end of 3 w, half of the mice from each group (n = 12/group) were euthanized, and the remaining mice (n = 12/group) were placed in standard mouse cages and allowed full, free reambulation (RA). During the 3 w of reambulation, RA+VIB and RA+SHAM groups continued to receive the daily vibratory or sham loading. Body mass of all mice was monitored daily for the duration of the experiment. Mice were injected with calcein (15 mg/kg, i.p.) 12 and 2 days prior to sacrifice. After overnight fasting, mice were sacrificed and serum was collected by cardiac puncture. Left tibiae were stored in 10% formalin for micro-computed tomography (µCT) and histomorphometry. Right tibiae and femurs were stored in cold PBS for flow cytometry.

While the experimental design of the first phase of this study was designed to determine whether vibrations applied both during disuse and reambulation provide benefits to cells and bone tissue, a preliminary follow-on experiment attempted to identify whether treatment with the low-magnitude vibratory signal was more influential during the disuse or reambulation period. In this second phase, matching the experimental protocol of phase I, two additional groups of male 7 w-old C57BL/6J mice were subjected to 3 w of hindlimb unloading followed by 3 w of reambulation. In the first group of phase II mice, 15 min of the daily vibratory stimulus described above was applied only during HU but not during RA (VIB-HU, n = 6). The second group of phase II mice received vibrations only during RA, but not during HU (VIB-RA, n = 6). Tibiae of mice in these two groups were *in vivo* µCT-scanned at baseline, after the 3 w disuse period and after the 3 w reambulation period.

### Micro Computed Tomography

Extracted tibiae of phase I mice were fixed in 10% formalin at 4°C [29] and transferred to a 70% ethanol solution in which the proximal tibia was scanned by µCT at a 12 µm isotropic voxel size (MicroCT40, Scanco AG, Switzerland). Under isoflurane anesthesia [30], the proximal tibiae of phase II mice were µCT-scanned at the same voxel size *in vivo* at baseline, after HU, and after RA (VivaCT40, Scanco AG). The µCT scanner only exposed the scanned region to radiation, thus minimizing the possibility of radiation effects on the tissue and bone marrow [31], [32]. For both *ex-vivo* and *in-vivo* scans, the metaphysis was defined as a 600 µm long region extending from the proximal tibial-fibular junction. Trabecular bone was separated from the surrounding cortex by an automated algorithm [33].

Software provided by the manufacturer of the scanner determined indices of bone morphology. For trabecular bone, trabecular bone volume (Tb.BV), trabecular bone volume fraction (BV/TV), trabecular connectedness (Conn.D), trabecular thickness (Tb.Th), and trabecular number (Tb.N) were quantified via previously established algorithms [34]. Trabecular tissue mineral density (Tb.TMD) was determined via calibrated phantoms [35] in the central region of the trabeculae to avoid edge artifacts [36]. Compared to sychotron based µCT, TMD may have been slightly underestimated but relative differences between samples are accurate [37]. For the cortical bone surrounding the trabecular volume of interest, the average cortical bone area (Ct.Ar), cortical bone thickness (Ct.Th), and bone marrow area (Ma.Ar) were computed.

### Histomorphometry

After µCT scanning, tibiae were dehydrated and embedded in PMMA. Coronal, 4 µm thick sections of the proximal tibia were cut (n = 6 per group and time point). Two non-consecutive slices were analyzed for dynamic indices of bone formation, including mineralizing surface (MS/BS), mineral apposition rate (MAR), and bone formation rate (BFR/BS). The remaining slices were decalcified and either stained with 1% toluidine blue (TB, 2 per bone) to determine osteoblast number (N.Ob) and surface (Ob.S), or stained for tartrate resistant acid phosphatase (TRAP, 2 per bone) to quantify osteoclast surface (Oc.S). All measurements were performed using Osteomeasure software (Osteomeasure, OsteoMetrics Inc., Atlanta, GA). Furthermore, the number of adipocytes (N.Adi) embedded in the marrow of the region of interest was measured based on the easily identifiable size and geometry of fat droplets without any specific stain [38]. The ratio of N.Ob to N.Adi (N.Ob/N.Adi) was determined as an indicator of cells having differentiated into an osteoblastic, rather than adipogenic lineage.

### Flow Cytometry

Flow cytometry measurements were acquired as detailed elsewhere [25]. Briefly, bone marrow was flushed from the right diaphyseal tibia and femur, strained in cold PBS and subjected to 1% Pharmlyse (BD Bioscience, San Diego, CA) to remove red blood cell contamination. Cells were then immediately stained with PE conjugated Sca-1 and FITC conjugated CD90.2 antigens (BD Bioscience) to detect progenitor marrow cell populations [39], [40]. Flow cytometry (FACScan, Becton Dickinson, San Jose, CA) counted the stained cells and quantified their size (forward scatter or FSC) and granularity (side scatter or SSC). Proportions of Sca-1 and CD90.2 positive cells to the total viable marrow cell population were reported (n = 6 per group and time point).

### ELISA

Collected blood volumes were centrifuged (4°C, 5000 rpm, 10 minutes) and serum was separated from blood cells. ELISA kits were used to measure serum concentrations of osteocalcin (Biomedical Technologies, Stoughton, MA), a protein implicated in bone formation and mineralization [41], osteopontin (R&D Systems, Minneapolis, MN), a protein associated with bone resorption [42], and insulin like growth factor (IGF-1, ALPCO, Salem, NH), a protein which anabolic effects can be impaired during disuse [43] and stimulated during reambulation [44] (n = 6 per group and per time point).

### Statistics

All data were presented as mean ± SD. Paired t-tests were used to compare the longitudinal changes in body mass within each group. Group means of cross-sectional data were compared by ANOVA and, if significant, a Student-Newman-Keuls (SNK) post-hoc test was applied. At any given time point, the two groups of phase II mice were compared to each other with unpaired Student's t-tests. Statistical significance was set at 5%. The statistical analyses were repeated with adjusting for differences in body mass as a covariate. However, adjusted data did not deviate significantly from the original comparisons and only unadjusted data were reported.

## Results

### Longitudinal changes in body mass

**Figure 1:**
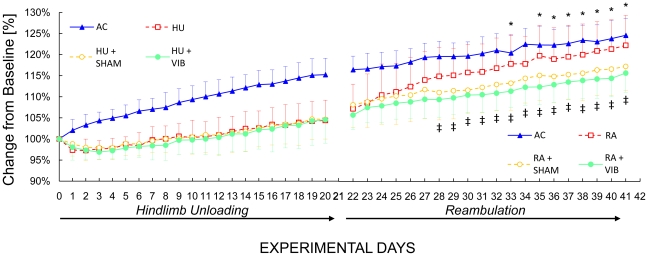
Longitudinal changes in body mass relative to baseline values. After 21 days of unloading, half of the mice from each unloaded group were released and allowed to re-ambulate. Age-matched controls: AC, solid triangles; hindlimb unloaded: HU, empty squares; sham loaded during unloading: HU+SHAM, empty circles; vibrated mice during unloading: HU+VIB, solid circles; reambulation: RA, empty squares; sham loaded during reambulation: RA+SHAM, empty circles; vibrated mice during reambulation: RA+VIB, solid circles. ‡: p<0.05 between RA+VIB and RA mice. *: p>0.05 between AC and RA mice (ANOVA and SNK).

During the first 3 w, body mass of AC mice increased continuously, while the body mass of the other three groups of mice (HU, HU+SHAM and HU+VIB) increased following a 1 w delay. At the end of the first 3 w experimental phase, AC, HU, HU+SHAM and HU+VIB mice had increased their body mass by 15%, 4%, 5% and 5% (all p<0.001) compared to baseline (Fig. 1). During reambulation, all groups continued to gain body mass, with RA mice showing the greatest increase resulting in a significantly greater (5–6%, p<0.05) body mass than of RA+VIB mice during the last 2 w of the reambulation period (Fig. 1). At the end of reambulation, AC, RA, RA+SHAM and RA+VIB mice had increased their body mass by 23%, 21%, 16% and 15% (all p<0.001) compared to baseline (Fig. 1).

### Bone marrow progenitor cells

**Figure 2:**
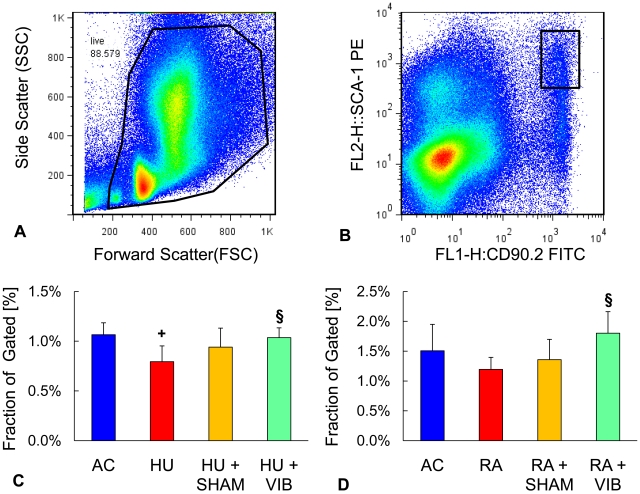
Flow cytometry of bone marrow cells. (**A**) Size and granularity distribution of total bone marrow cells. Region of interest was selected to include single and viable cell populations. (**B**) Fluorescence of cells that were positive for SCA-1 and CD90.2 surface antigens according to fluorescence intensity. Ratio of SCA-1 and CD90.2 positive cells to total gated cells (**C**) after 3 w and (**D**) after 6 w of the experiment. Data are mean ± SD. Groups were compared by ANOVA followed by SNK. Letters denominate significant (p<0.05) differences between groups: **+** any group different from AC; § HU+SHAM (RA+SHAM) or HU+VIB (RA+VIB) different from HU (RA); # HU+VIB (RA+VIB) different from HU+SHAM (RA+SHAM).

Flow cytometry measurements were assessed based on the scatter profile of all marrow cells (Fig. 2). Upon elimination of cell debris according to size and granularity, events that were positive for SCA-1 and CD90.2 antibodies were collected for viable cells (Fig. 2). After 3 w, the population of progenitor cells was 25% (p = 0.02) smaller for HU than for AC mice. In contrast, the population of progenitor cells for HU+VIB mice was 30% (p = 0.02) larger compared to HU mice (Fig. 2). After disuse followed by 3 w of ambulation, RA+VIB mice had a progenitor population that was 51% (p = 0.03) larger compared to RA mice while the other groups were not significantly different from each other (Fig. 2).

### Bone morphology after disuse and reambulation

**Figure 3:**
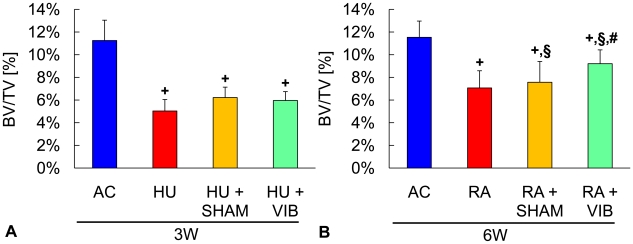
Bone morphology in the metaphysis of the proximal tibia. Trabecular bone volume fraction (BV/TV) of the proximal tibia in (**A**) age-matched controls (AC), hindlimb unloaded (HU), sham loaded (HU+SHAM), and vibrated (HU+VIB) mice after 3 w of disuse and (**B**) age-matched controls (AC), reambulation (RA), sham loaded (RA+SHAM), and vibrated (RA+VIB) mice after 3 w of disuse followed by 3 w of reambulation. Data are mean ± SD. Groups were compared by ANOVA followed by SNK. Letters denominate significant (p<0.05) differences between groups: **+** any group different from AC; § HU+SHAM (RA+SHAM) or HU+VIB (RA+VIB) different from HU (RA); # HU+VIB (RA+VIB) different from HU+SHAM (RA+SHAM).

Three weeks into the experiment, all disuse groups (HU, HU+SHAM and HU+VIB) had a smaller (−55%, −45% and −47%, all p<0.001) BV/TV in the proximal tibia compared to age-matched controls (Fig. 3). The group differences in BV/TV were accompanied by similar differences in micro-architectural trabecular parameters except for tissue mineral density which was not different between any of the groups (Table 1). No significant differences in trabecular morphology were observed between groups subjected to unloading.

**Table 1:** Trabecular and cortical bone morphology at the tibial proximal metaphysis in age-matched controls (AC), hindlimb unloaded (HU), sham controls (HU+SHAM), and vibrated mice (HU+VIB) after the 3 w disuse period, or age-matched controls (AC), reambulation (RA), sham controls (RA+SHAM), and vibrated mice (RA+VIB) after 6 w.

		AC	HU	HU+SHAM	HU+VIB
**3 W**	**Tb.BV [mm^3^]**	0.14±0.03	0.05±0.01^+^	0.07±0.01^+^	0.06±0.01^+^
	**Conn.D [1/mm^3^]**	120±41	14±11^+^	29±16^+^	23±12^+^
	**Tb.N [1/mm]**	6.08±0.35	4.58±0.44^+^	4.79±0.37^+^	4.80±0.20^+^
	**Tb.Th [µm]**	36.7±1.3	33.9±2.7^+^	34.9±2.2^+^	34.9±1.8
	**Tb.TMD [mgHA/ccm]**	793±26	798±23	803±17	808±19
	**Ct.Ar [mm^2^]**	0.59±0.07	0.51±0.04^+^	0.54±0.04^+^	0.53±0.04^+^
	**Ct.Th [µm]**	85.4±7.5	76.0±6.6^+^	78.9±7.3^+^	77.2±7.8^+^
	**Ma.Ar [mm^2^]**	1.75±0.15	1.50±0.12^+^	1.55±0.11^+^	1.56±0.08^+^

		AC	RA	RA+SHAM	RA+VIB
**6 W**	**Tb.BV [mm^3^]**	0.12±0.02	0.07±0.02^+^	0.08±0.02^+^	0.10±0.02^+,^ §,c
	**Conn.D [1/mm^3^]**	109±25	30±14^+^	35±20^+^	56±20^+,^ §,c
	**Tb.N [1/mm]**	5.73±0.25	4.54±0.39^+^	4.70±0.53^+^	5.03±0.36^+,^ §,c
	**Tb.Th [µm]**	39.0±3.0	39.5±2.5	39.3±2.9	40.2±3.3
	**Tb.TMD [mgHA/ccm]**	795±12	838±17^+^	835±13^+^	822±17^+,^ §,c
	**Ct.Ar [mm^2^]**	0.66±0.06	0.62±0.05	0.61±0.07	0.62±0.06
	**Ct.Th [µm]**	96.9±10.4	94.4±7.7	92.1±9.7	91.0±11.8
	**Ma.Ar [mm^2^]**	1.52±0.12	1.47±0.10	1.45±0.13	1.60±0.14§,c

Data are mean ± SD. Groups were compared by ANOVA followed by SNK. Letters denominate significant (p<0.05) differences between groups: **+** any group different from AC;

§HU+SHAM (RA+SHAM) or HU+VIB (RA+VIB) different from HU (RA); **#** HU+VIB (RA+VIB) different from HU+SHAM (RA+SHAM). Tb.BV: trabecular bone volume; Conn.D: connectedness; Tb.Th: trabecular thickness; Tb.N: trabecular number; Tb.TMD: trabecular tissue mineral density; Ct.Ar: cortical bone area; Ma.Ar: marrow area.

Mice that received the mechanical stimulus during the 3 w disuse and 3 w reambulation period (RA+VIB), had 22% (p = 0.003) and 30% (p = 0.01) greater BV/TV than RA and RA+SHAM groups at the end of the reambulation phase. However, the vibratory stimulus failed to fully restore trabecular bone within the short 3 w reloading period, as indicated by the 20% difference (p<0.001) in BV/TV between RA+VIB and AC mice (Fig. 3). The application of low-level vibrations provided benefits not only to the quantity of metaphyseal trabecular bone but also to its intricate morphology. Micro-architectural parameters including trabecular connectedness and the number of trabeculae displayed group differences that were similar to those reported for BV/TV except trabecular thickness which, upon reambulation, was fully restored to age-matched control levels in all disuse groups (Table 1). At the end of the reambulation period, trabecular bone of the disuse groups had a greater tissue mineral density than that of age-matched controls, a difference that reached 5% for RA and RA+SHAM mice and 3% for RA+VIB mice (all p<0.001).

For cortical bone surrounding the trabecular bone described above, 3 w of unloading of any of the three experimental groups resulted in cortical bone properties that were significantly deteriorated compared to those of age-matched control groups (Table 1). There were no significant differences between the unloaded groups. Upon reambulation, neither cortical area nor thickness were different between any of the four groups. Bone marrow area was 11% (p = 0.02) and 9% (p = 0.03) greater in HU+VIB than in RA+SHAM and RA mice.

### Histology

**Figure 4:**
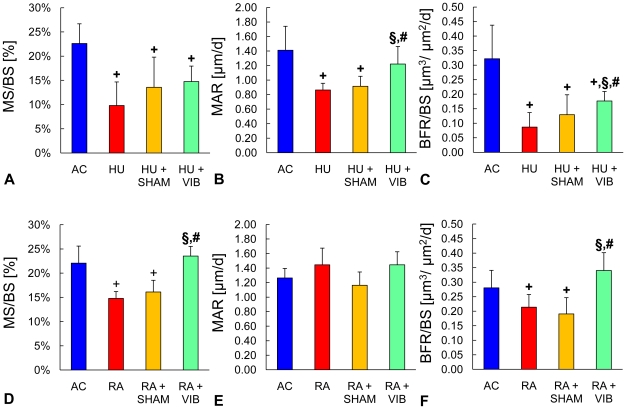
Bone formation in the metaphysis of the proximal tibia. Static and dynamic indices of bone formation at trabecular bone surfaces of (**A-C**) age-matched controls (AC), hindlimb unloaded (HU), sham loaded (HU+SHAM), and vibrated mice (HU+VIB) after the initial 3 w phase and of (**D-F**) age-matched controls (AC), reambulation (RA), sham loaded (RA+SHAM), and vibrated mice (RA+VIB) at the end of the 6 w experimental phase. Data are mean ± SD. Groups were compared by ANOVA followed by SNK. Letters denominate significant (p<0.05) differences between groups: **+** any group different from AC; § HU+SHAM (RA+SHAM) or HU+VIB (RA+VIB) different from HU (RA); # HU+VIB (RA+VIB) different from HU+SHAM (RA+SHAM).

Disuse for 3 w resulted in MS/BS that were 57% smaller (p<0.001) in HU mice than in AC mice (Fig. 4). Compared to HU mice, neither HU+SHAM, nor HU+VIB mice showed significant differences in MS/BS at the end of the 3 w period. Dynamic indices of bone formation were also reduced during disuse as evidenced by 39% and 73% (both p<0.001) smaller MAR and BFR/BS for HU than for AC mice. Vibrated mice had 41% greater (p = 0.03) MAR than HU mice, an improvement which HU+SHAM mice failed to show (Fig. 3). As a result, vibrations increased BFR/BS two-fold (p<0.001) compared to HU mice but still remained 45% smaller (p = 0.002) compared to AC. After 3 w of reambulation, smaller MS/BS (33%, p<0.001) were found in RA mice compared to AC (Fig. 4). On the other hand, RA+VIB mice showed 59% (p<0.01) greater MS/BS and BFR/BS than RA mice during reambulation (Fig. 4).

**Figure 5:**
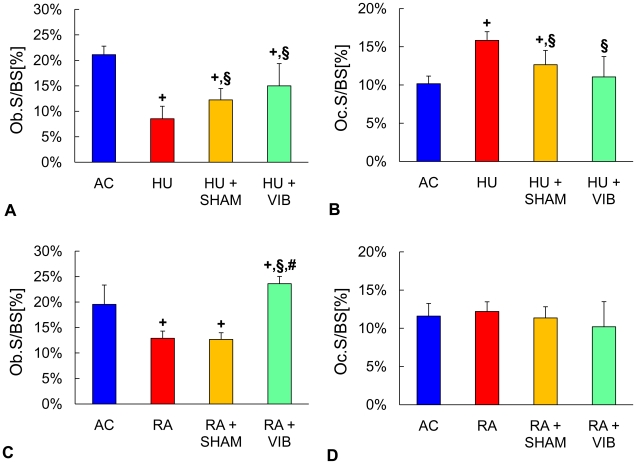
Relative cell numbers in the metaphysis of the proximal tibia. Indices of osteoblast (Ob.S/BS) and osteoclast (Oc.S/BS) number of (**A,B**) age-matched controls (AC), hindlimb unloaded (HU), sham loaded (HU+SHAM), and vibrated mice (HU+VIB) after the initial 3 w phase and of (**C,D**) age-matched controls (AC), reambulation (RA), sham loaded (RA+SHAM), and vibrated mice (RA+VIB) at the end of the 6 w experimental phase. Data are mean ± SD. Groups were compared by ANOVA followed by SNK. Letters denominate significant (p<0.05) differences between groups: **+** any group different from AC; § HU+SHAM (RA+SHAM) or HU+VIB (RA+VIB) different from HU (RA); # HU+VIB (RA+VIB) different from HU+SHAM (RA+SHAM).

Compared to trabecular toluidine blue stained sections of age matched controls, disuse mice without intervention had 60% smaller (p<0.001) Ob.S/BS (Fig. 5). Disuse combined with 15 min/d of normal activities (HU+SHAM) caused 44% greater (p = 0.03) Ob.S/BS as compared to HU mice. Vibrated mice (HU+VIB), however, showed 76% greater (p = 0.002) Ob.S/BS than HU mice. After the combined disuse and reambulation periods, RA mice had 34% smaller (p<0.001) Ob.S/BS than AC mice (Fig. 5). There were no significant differences in Ob.S/BS between RA+SHAM and RA mice (Fig. 5). In contrast, RA+VIB mice had 83% greater (p<0.001) Ob.S/BS than RA mice and 21% greater (p = 0.01) Ob.S/BS than AC mice. Indicating a shift in commitment of marrow progenitor cells from osteoblastogenesis to adipogenesis, the proportion of osteoblasts to adipocytes (N.Ob/N.Adi) for HU, HU+SHAM and HU+VIB levels was 89% (p = 0.002), 89% (p = 0.004) and 66% (p = 0.005) smaller than those in AC mice after 3 w of disuse (Table 2). Upon reambulation, N.Ob/N.Adi was 235% greater (p = 0.04) in RA+VIB than in RA+SHAM mice (Table 2).

**Table 2:** Indices of osteoblasts, adipocytes and serological factors measured in the proximal tibia or the serum in age-matched controls (AC), hindlimb unloaded (HU), sham controls (HU+SHAM) and vibrated mice (HU+VIB) after the 3 w disuse period, or age-matched controls (AC), reambulation (RA), sham controls (RA+SHAM) and vibrated mice (RA+VIB) after 6 w.

		AC	HU	HU+SHAM	HU+VIB
**3 W**	**N.Ob/N.Adi [-]**	9.8±4.9	1.8±1.4^+^	1.4±1.4^+^	1.8±1.3^+^
	**Serum OC [ng/ml]**	36±5	20±7^+^	14±4^+^	16±4^+^
	**Serum OPN [ng/ml]**	216±73	276±54	364±110^+^	373±131^+^
	**Serum IGF-1 [ng/ml]**	252±79	165±86	162±50	274±139

		AC	RA	RA+SHAM	RA+VIB
**6 W**	**N.Ob/N.Adi [-]**	3.1±1.8	1.2±0.6	1.8±1.3	4.0±1.8§
	**Serum OC [ng/ml]**	21±10	20±11	15±5	18±4
	**Serum OPN [ng/ml]**	219±51	233±123	303±86	186±68
	**Serum IGF-1 [ng/ml]**	323±105	284±88	258±58	399±48

Data are mean ± SD. Groups were compared by ANOVA followed by SNK. Letters denominate significant (p<0.05) differences between groups: **+** any group different from AC;

§HU+SHAM (RA+SHAM) or HU+VIB (RA+VIB) different from HU (RA); # HU+VIB (RA+VIB) different from HU+SHAM (RA+SHAM). N.Ob: osteoblast number; N.Adi: adipocyte number; OC: osteocalcin; OPN: osteopontin.

Bone resorption, as measured by osteoclast surface relative to trabecular bone surface (Oc.S/BS), was 56% greater (p<0.001) in HU mice compared to AC (Fig. 5). Interruption of disuse either by weight-bearing or weight-bearing and vibrations affected Oc.S/BS as evidenced by 20% (p = 0.005) or by 30% (p<0.001) smaller values. Upon reambulation Oc.S/BS did not show any significant difference between groups (Fig. 4).

### Systemic factors of bone metabolism

After disuse, serum levels of osteocalcin was smaller in HU, HU+SHAM, and HU+VIB mice than in AC mice (44%, 60% and 54%, all p<0.001, Table 2). No significant differences in osteocalcin levels were detected between groups upon reambulation (Table 2). Osteopontin levels were 68% (p = 0.04) and 73% (p = 0.04) greater in HU+SHAM and HU+VIB mice than in AC mice with no significant differences between HU+VIB and AC mice (Table 2). Upon reambulation, similar to osteocalcin, osteopontin levels did not show any significant differences between groups (Table 2). No significant group differences in IGF-1 serum levels were detected after 3 w and 6 w (Table 2).

### Comparing the influence of vibration during disuse versus recovery

**Figure 6:**
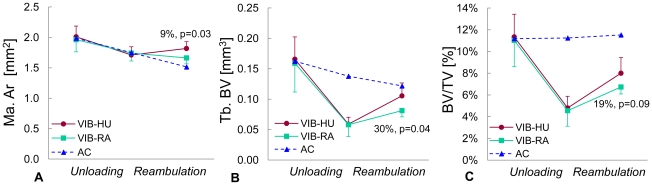
Comparison between vibrations applied during unloading vs reambulation. Longitudinal changes in (**A**) bone marrow area (Ma.Ar), (**B**) trabecular bone volume (BV), and (**c**) trabecular bone volume fraction (BV/TV) of the tibial metaphysis in mice that either received vibrations *only* during the unloading period (VIB-HU, n = 6) or *only* during reambulation (VIB-RA, n = 6). Open triangles correspond to-cross-sectional data of age-matched control mice (n = 12). Relative differences and p-values are given for the comparison between VIB-HU and VIB-RA mice.

In a preliminary follow-on study, to determine the relative contributions of the vibratory stimulus to bone morphology during disuse vs reambulation, vibrations were applied *only* during the 3 w disuse period (VIB-HU), or *only* during the 3 w reambulation period (VIB-RA). Morphological evaluations were performed longitudinally at baseline, 3 w and 6 w. No significant difference for Ct. Ar was found between groups at any time point. Compared to mice that received vibrations only during reambulation (VIB-RA), mice that received vibrations only during disuse (VIB-HU) had similar bone morphology at baseline and after 3 w of disuse (Fig. 6). Upon 3 w of reambulation, however, VIB-HU mice had a 30% (p = 0.04) greater trabecular bone volume and a 9% (p = 0.03) greater Ma.Ar (Fig. 6).

## Discussion

The ability of high-frequency, low-level vibrations to protect and augment cellular indices, tissue quantity, and trabecular morphology was investigated during disuse and reambulation in the tibia of young mice. Disuse for 3 w eroded trabecular bone morphology in the proximal tibia, and neither 15 min/d of weight-bearing nor the application of low-magnitude mechanical signals were able to prevent this loss. In contrast to the lack of a response at the level of the tissue, mice subjected to mechanical loading preserved, at least in part, their bone marrow based osteoprogenitor cells during disuse. Following the disuse period with three weeks of reambulation, the enhanced population of osteoprogenitor cells in the bone marrow of mechanically stimulated mice coincided with a greater degree of recovery of trabecular bone compared to non-stimulated mice. Supporting the hypothesis that the ability of bone to recover will correlate with the status of the bone marrow cell population, subsequent experiments showed that vibrations applied during disuse, rather than reambulation, were more effective in altering bone morphology upon reambulation. Combined, these data indicate that the application of high-frequency, low-magnitude mechanical stimuli during disuse may provide a benefit towards the regeneration of lost bone tissue, a critical achievement despite their inability to retain bone tissue challenged with disuse.

As apparent from the changes which occurred in the tibia over the course of the protocol in age-matched controls, the male C57BL/6J mice used in this study had reached peak trabecular bone mass. While the lack of regional *trabecular* bone growth eliminated the effect of growth as a potentially confounding factor, mice were still adding cortical bone mass and extrapolations to a primarily quiescent adult skeleton cannot be made. In adult rodents, vibrations applied at similar levels as in this study can stimulate trabecular bone formation [27], [45]. During disuse, the mechanical signal normalized the levels of bone formation to those of age-matched controls [22] but was not able to prevent the loss of tissue [23]. At the cellular level, low-level vibrations protected marrow stem cells against aging [46]. Thus, similar to young animals, marrow cells and bone's formative processes in the adult skeleton are clearly capable of sensing and responding to the vibratory signal. However, differences in mediating factors such as the level of bone turnover, metabolic rate, or composition of the progenitor cell pool emphasize that the relevance of our current data to an aging population needs to be addressed through additional experiments in the future. The interpretation of results from the flow cytometry measurements was based on only two markers expressed by stem cells with osteogenic potential [39], [40]. As such, this population was enriched but not exclusive for mesenchymal stem cells. Consequently, the flow cytometry data should be considered preliminary and eventually confirmed by primary marrow cells cultured *in vitro*. Finally, relative differences between groups need to be interpreted within the context of the chosen time points and no conclusions should be drawn regarding the long-term magnitude of tissue recovery in any of the groups.

Similar to previous reports [7], [47], mechanical unloading of the hindlimbs reduced the quantity of surface osteoblasts, mineralizing surfaces and mineral apposition rates. This suppression of bone formation, in conjunction with an increase in the number of osteoclasts, conspired towards an overall net loss of bone. Interrupting disuse by 15 minutes of weight-bearing activities attenuated the decline in bone formation and increase in osteoclast number but not the loss of bone tissue. That brief ambulatory periods proved insufficient to maintain bone morphology during unloading is consistent with the failure of weight-bearing [22] or rigorous exercise [1], [48] to stem the osteopenia caused by extended bedrest or weightlessness. During disuse, a daily superposition of low-magnitude vibrations upon the 15 minutes of weight-bearing did not preserve the trabecular structure either but stimulated the cellular status such that they remained similar to those measured in the weight-bearing control cohort. Importantly, these low-magnitude mechanical signals also increased the uncommitted stem cell pool in the marrow, protected mineral apposition rates, and increased bone formation rates.

Similar to humans recovering from spaceflight [49], chronic debilitating injury [50], or bedrest [2], disuse and sham control mice showed only hampered recovery of trabecular bone morphology upon reambulation. We hypothesize that this observation was a direct consequence of the disuse induced suppression of the osteoprogenitor cell population. Previous data from cultured bone marrow cells in rats subject to disuse and then allowed to ambulate demonstrated that the inability to reestablish bone formation rates was modulated by reduced osteoblast recruitment [8]. Our results support the conclusion that disuse causes a suppressed osteoprogenitor cell population as well as a diminution of osteoblast recruitment during early reambulation. Thus, protecting the regenerative potential of the bone marrow cell population during the challenge of disuse may be critical for tissue recovery during reambulation.

Superimposing the vibratory signal upon 15 min/d of habitual loading activities prior to full reambulation protected osteoprogenitor populations and mature osteoblasts. Upon restoration of weight-bearing, vibrated animals were able to more quickly increase bone formation rates as well as the quantity and morphology of trabecular bone. This greater potential for restoration of trabecular bone quantity and morphology is supported by previous data that demonstrated the ability of low-level vibrations to shift the preference of bone marrow progenitor cells towards osteoblastogenesis over adipogenesis [25]. Consistent with this data, vibrations increased the proportion of bone cells to fat cells within bone marrow, avoiding the bias towards adipogenesis at 6 wk. Further, those mice that received mechanical signals showed a smaller increase in body mass during reambulation, similar to previous studies in which whole body vibrations suppressed adipogenesis in normally ambulating mice and rats [51], [52]. While this may indicate that brief periods of either weight bearing or low magnitude mechanical signals during disuse decreased the likelihood of stem cells to steer towards adipogenesis, the combination of increased body mass and deteriorated trabecular morphology would inevitably compound the risk of fracture in bone. These data also emphasize that future studies will need to identify the outcome of mechanically altered marrow composition across a range of tissues, including altered levels of fat and muscle volume at peripheral and internal locations.

The reambulation period in this study was relatively short and at its completion, trabecular bone quantity of the mechanically stimulated mice was still lower compared to age-matched controls. However, greater indices of bone formation in mechanically stimulated mice than in untreated or sham controls suggested a trend of a faster and/or more complete recovery towards levels seen in age-matched controls. After three weeks of reambulation, all three experimental groups had higher levels of tissue mineral density than age-matched controls but TMD was smaller in vibrated mice than in the other two experimental groups. As tissue age is a critical determinant of TMD [35], higher bone formation rates in mechanically stimulated mice than in SHAM or HU/RA mice over the 6 w protocol may have caused a lower ratio of old and denser tissue to new and less dense tissue.

In summary, brief daily periods of whole body vibrations delivered at a relatively high frequency but low magnitude did not prevent the deterioration in bone morphology caused by disuse, yet significantly improved the osteogenic potential of bone marrow cells, the population ultimately responsible for recovery of the bone structure. The augmented regenerative response of trabecular bone during reambulation in mechanically stimulated mice may have resulted from a more viable, osteogenic stem cell population, as bone was more responsive to the mechanical signals applied during disuse than during reambulation. Our data may also indicate that pharmaceutical interventions which may or may not preserve bone structure during disuse [53]–[55], may be limited in their ability to regenerate bone tissue and maintain skeletal health if they don't address the collapsing of the osteoprogenitor pool. If confirmed that preserving or increasing the osteogenic potential of bone marrow cells is critical for bone regeneration, the application of high-frequency mechanical signals during space flight, bedrest, or immobilization may accelerate recovery upon restoration of weight bearing.
